# Retrospect of the Medical Sciences

**Published:** 1843-11-25

**Authors:** 


DR. ASHWELL ON IRRITABLE UTERUS.

"Irritable uterus" is the next affection to which the author's attention is directed, and in this section we find much that is valuable in reference to the diagnosis of this form of disease from the many others with which it is frequently confounded. The correctness of the term "irritable uterus" is questioned, and with much justice; before the writings of the late Dr. Gooch, whose admirable description of the disease first drew sufficient attention to it, physicians were accustomed to believe in the occurrence of inflammation as the cause of the frequently urgent symptoms grouped under the head of irritability. An error is now far too common of believing in the frequent existence of irritability of the uterus rather than of inflammation, and the practice pursued in accordance with such views commonly falls short of its intended object, no benefit results, and the disease runs on, its severity alone ameliorated by the frequent exhibition of sedatives. The symptoms of this affection, stripped of the obscurity which their existence in persons of hysteric diathesis often throws around them, are, in fact, distinctly those characteristic of inflammatory action.

"That the disease in question (says Dr. Ashwell) should be regarded as a modified inflammation of the cervix uteri, is a view in accordance not only with symptoms, but with the results of the most successful treatment. It is difficult to understand that there shall be redness, which I have several times seen by the speculum, heat, permanent pain, and the tenderness of the neck of the uterus, a glandular part, without believing that its vascular and nervous structures have undergone some change. Judging, also, from the marked relief afforded by cupping, leeching, aperients, and spare diet, what more tenable and satisfactory conclusion can be arrived at than that the so-called irritable uterus is dependent on subacute or chronic inflammation—a position, the truth of which is fully substantiated by those changes of structure which, although slowly, and not till after many years have, nevertheless, occurred, in cases which till then were regarded and treated as examples of irritable or neuralgic disease."

Several interesting cases illustrative of the efficacy of anti-phlogistic treatment in this class of affections is given, more especially of the employment of local depletion.—*Prov. Medical Journal*, Sept. 16, 1843.

### RETROSPECT OF THE MEDICAL SCIENCES.

### ON BRONCHIAL BLENORRHŒA, ETC., CONSIDERED IN CONNECTION WITH RHEUMATISM.

BY R. R. CHEYNE, SURGEON.

My observation has been for some time past turned upon a well marked form of what may be called bronchial blenorrhœa, which is clearly dependent upon the metastasis of rheumatism, or at least, upon the rheumatic diathesis and which is as clearly unconnected with bronchitis. As this affection, as far as I know, has not been described, I will sketch a case which, in most respects, may be considered a type of the whole class; and at the same time, I will point out the remedies which, in my hands, have been strikingly and uniformly beneficial.

Case.—Mr. B., æt. about 60, a strong healthy man, whose occupation exposed him, for many hours daily, to a very high temperature, and subjected him constantly to profuse perspirations, became in June last, affected with troublesome cough, without expectoration or dyspnœa. His pulse was natural and skin cool; his appetite was deficient; he became weak and out of spirits, perspired profusely at night, and passed little urine, which was loaded with the lithates. At the end of a week he began to expectorate large quantities of muco-purulent fluid, without relief to his cough; he got weaker and lost flesh; so much so, that his friends feared consumption, to which view of the case, however, auscultation gave the negative, inasmuch as, at first, no morbid condition whatever could be detected, and it was only when free secretion was taking place, that rhonchi could be heard shifting about in every ramification of the air-tubes. Besides, percussion elicited a clear sound over the whole of the chest. Now, the treatment of the patient, during a fortnight, consisted in the application of a blister, the use of mild doses of mercury and ipecacuanha until the gums were slightly touched, and of occasional opiates at night to relieve the cough; at the same time the diet was strictly regulated. This plan, which was steadily pnrsued, failed in controlling the symptoms. My patient was then sent a few miles into the country, where I continued to visit him daily; and (as the enormous expectoration was producing great debility, and there was a total freedom from fever), I changed the treatment, and prescribed quinine with iron, and a liberal diet. Nevertheless, the disease still defied the remedies, after three weeks' duration. I must confess I was now puzzled, until one morning I found my patient complaining of an attack of severe pain in the ankle-joint, which was not accompanied by either swelling or redness; and in addition, I then learnt that he had been for a year or two occasionally subject to pain and slight puffy swelling of the right knee-joint, though never to the degree of confining him to his house. He now, also, recalled to mind, that he had felt great weakness in the knee, but not in the ankle, just before his present illness. These invaluable hints of the true origin of the complaint, I was not slow to act upon. I at once ordered for him a pill composed of two grains of the acetous extract of colchicum, and three grains of the compound extract of colocynth, to be taken at night as an aperient; and, in the course of twenty-four hours, a mixture containing of Iodide of Potasium ℈j., of liquor Potassæ, and tincture of Henbane aa. ʒj. in Mist. Camph. ℥vj. He was also directed to place his feet every night and morning, in a hot mustard and water bath.

In so short a period as three days, under these remedies, the bronchial blenorrhœa was reduced to half its former quantity, and at the end of a week it had ceased altogether. Soon afterwards his cough left him; and his health and strength were rapidly restored.

Two facts may, I think, be fairly deduced from the history of this case; viz. first, that a peculiar irritation of the bronchial mucous membrane, with profuse secretion, constituting a distinct kind of bronchial blenorrhœa, may be induced by the rheumatic diathesis; and secondly, that iodide of potassium (as in the above formula) is, undoubtedly a medicine of astonishing power over this state of the mucous membrane. I may add, that colchicum in my hands, has frequently proved to be of greatly inferior efficacy.

I can, certainly, come to no other conclusion but that the description I have given of the above disease cannot, with any regard to accuracy, be identified with that of any form of bronchitis, unless the latter term be used without the least reference to the actual symptoms.

Having thus endeavored to show, by analysis, the existence of a rheumatic form of bronchial blenorrhœa, I will now, in order to make the evidence as conclusive as I can, examine the subject by a method like that of synthesis, in briefly alluding to a class of morbid phenomena, often excited in another tract of mucous membrane; viz. the genito-urinary, which phenomena clearly evince the close connection that obtains between either irritation, or simple discharge from that membrane, and the development of rheumatism, in individuals who possess the rheumatic diathesis. For example, gonorrhœa, urethral blenorrhœa, and even the irritation and discharge sometimes brought on by the passage of a bougie (as I have again and again witnessed), may be excitants of rheumatism. Lucorrhœa, also, is often attended by the same complication, especially in the form of neuralgia, to which young women are extremely predisposed. Lastly, I may observe, that, for the supervention of rheumatism, it is by no means necessary that this irritation, and increased secretion, be of a specific nature, as in gonorrhœa. It was with reference to what has been named gonorrhœal rheumatism, that Swediaur, who first noticed it, asks the question, whether, in such cases, gonorrhœa is not rather arthritic than syphilitic; and I am, myself, almost disposed to agree with Richter, and others, who attribute most forms of these discharges to transferred rheumatic action.—*Lon. Med. Gazette*, Sept. 15, 1843.

---

### EXTRACT OF INDIAN HEMP.

Mr. Savory has communicated to the "Pharmaceutical Journal" the following formula for preparing the extractum cannabis Indicæ:—Take of gunjah (finely bruised), four pounds, avoirdupois; rectified spirit (0.838), five gallons, old m.; macerate the gunjah in two gallons of the spirit for seven days, then strain off and add one gallon more of the spirit; let this stand four days, and strain; mix the two tinctures and filter; then boil the hemp in the remaining two gallons for fifteen minutes, and filter *while hot*. Let all the tinctures be mixed; then distil off the spirit and evaporate the remainder in a water bath to the consistence of an extract. Produce—twelve ounces.

*Prov. Med. Journal.*

VITAL STATISTICS OF SHEFFIELD.

One of the most prosperous trades in Sheffield is the silver and plated manufacture. Until the last two years it has not shared the distress experienced by all engaged in making cutlery. The workmen belonging to this branch amount to 400, and form several unions, the restrictive laws of which are effective, even in times of bad trade. The earnings of men vary from 18 to 42 shillings, and of women from 8 to 15 shillings a week.

The workmen in this manufacture are sober, intelligent, steady, and are, or were, in tolerable circumstances. This is attributable to various causes. First, the rule of the trades' union, which prohibits the masters from taking apprentices, has reduced the number of workmen, formerly superabundant. Secondly, the expensive materials and tools used in this trade, prevent that sudden conversion of men into masters which is so common in so many other occupations. Thirdly, children are rarely employed in this trade, much under fourteen years of age. Fourthly, the workmen are not liable to be thrown out of employment by machinery. This occupation is neither particularly detrimental to health, nor conducive to longevity. In the Silversmiths' Benefit Society, the mean age attained by those who die above the age of 40 is 59.12; while among the working classes, generally, of Manchester, Leeds, and Liverpool, it is 58.97; so that the advantage in favor of the silversmiths is slight indeed. In rural districts the mean age attained by persons above 40, is 68.76. What a difference!

Next come the *saw manufacturers.*

"The workmen in this branch of trade are, perhaps, in no degree inferior in intelligence, sobriety, and general good conduct, to those in the manufacture of which we have just treated. They have both, equally, their respective unions, which regulate wages, the introduction of apprentices, and which, in time of sickness, afford a weekly allowance."

In the branch called *par excellence* saw making, there are 208 journeymen, of whom about 20 are not in the union; 130 boys—being, indeed, more than the rules of the union allow; and about one woman or girl to every eight men.

Nine-tenths of the men are in sick clubs; nineteen out of twenty, can read and write; and their average wages are 28 shillings a week, the extremes being 24 shillings and 45 shillings.

The *saw grinders* are fine healthy men, who generally live in the country, and often add the cultivation of a plot of ground, or even a small farm, to their mechanical occupation. They are peculiarly liable to accidents, and of 42 saw grinders who have died since 1821, 5 were killed by the breaking of stones. Dr. Holland gives a list of 13 accidents, being a part of those which have happened to 78 living members in the union. Most of them are very serious, *e. g.* "3. Drawn over the stone; severely hurt; in bed nine months." "9. Arm severely lacerated; lame three months."

The *edge-tool makers* are well paid, but are a less intelligent class than those previously mentioned. The number of *foremen* and *strikers* is equal in this trade; the former are said to earn, on the average, £1. 14s., and the latter £1. 2s. a week.

The account is furnished by a manufacturer, and, though no doubt accurate as far as it goes, is defective in an important point. We do not learn from it how many months in the year the men are at work. The prices have been strictly enforced, even during the last three years, but for what portion of that time have the men obtained them?

Three-fourths of the workmen in this branch are in sick clubs; but only one among five adults can read and write. Trades that require strong arms rather than acute heads, will be favorable rather to sensuality than intelligence; a blacksmith drinks more than he reasons; and Dr. Holland finds that the forgers of Sheffield have big occiputs, but low, retreating foreheads.

The spring knife makers are badly paid, and badly educated. "A few superior workmen may earn from 30s. to 40s. per week. In the first manufactories of the town the average is from 16s. to 25s. But in many of the inferior manufactories, the workmen are receiving no more than 12s. or 16s.

Moreover, when Dr. Holland was writing, in June 1843, this scale of wages was very greatly reduced.

This branch is not in union; not above half of the adults can read, and not one-fourth moderately well; about two-thirds only of the adults are in sick clubs.

Two circumstances combine to depress wages in this department. The most important is the smallness of the capital required to set up as a cutler. A few pounds are sufficient. The other one is the facility which this branch offers for the employment of children at an early age.

The *file trade*, as regards the circumstances of those employed in it, holds a middle place between the silversmiths and saw-makers, who are above, and the spring knife cutlers, who are below them.

The branch of the file trade called *forgers*, consists, like the edge-tool makers, of foremen and strikers; but the profits are more equally divided between the two classes of workmen in the present instance; a foreman averaging £1. 12s. 10d. a week, and a striker £1. 6s. 9d.

As to *file-cutters*, by reference to thirty books of file-cutters, men of *steady* habits, each having the assistance of a boy, the average per week was found to be £1. 18s. 6d. Eighty per cent. of the adults can read, and seventy can write. The proportion among the boys is rather greater; which may either show that education is making way, or that a certain number of adults forget their little scholastic learning.

The *fork-grinders* carry on the most unwholesome trade in the kingdom. Fork-grinding is always performed on a dry stone, and the clouds of stony and metallic dust produced in the process, clog up the respiratory organs, and cause grinders' asthma, a disease which carries off its victims in a majority of cases, before the age of thirty!

The better the state of a given trade in Sheffield, says Dr. Holland, the smaller is the proportion of minors employed in it. Thus in the silver-plated branch, the minors are to the adults as 16 to 100; and among the edge-tool makers, as 12½ to 100. In the spring knife branch the per centage is about 25; but among the fork-grinders the minors actually outnumber the adults, as there are a 100 boys to only 97 men!

Of the ninety-seven, about thirty are at this moment suffering from grinders' asthma. It is remarkable that in this destructive trade the men are ill paid, but Dr. Holland does not say what their wages are. Nor is their education superior to their condition; for of the 197 men and boys, one hundred and nine can only read, and 69 can write.—*London Medical Gazette*, Sept. 8th, 1843.

---

SPUTA IN BRONCHITIS. BY DR. SIMON, OF BERLIN.

In consequence of a very violent attack of bronchitis, the patient expectorated a purulent mucus, in which Dr. Schönlein detected a white, filiform substance, which fell to the bottom; when this was placed in water, it was observed that several long, fine threads, ramifying as from a tree, shot off from the trunk of the white, filiform substance, and ran off in extremely fine terminations, which, on gently moving the vessel in the water, floated to and fro; on placing these in acetic acid, they swelled and become changed into a transparent jelly, which was in a great measure dissolved after digesting for a considerable time; ferrocyanuret of iron produced in this solution a precipitate; under the microscope these threads exhibited the structure of coagulated fibrine. There is no doubt that in this case, in consequence of the inflammation of the bronchi and of their finest ramifications, plastic lymph was effused in the latter, which was expectorated in the coagulated state.—*Provincial Medical Journal, from Beiträge Zur Physiologischen und Pathologischen, Chemie und Mikroscopie.*

---

### FORMULA FOR OPODELDOC.

In the October No. of the Journal of Pharmacy, Mr. Augustine Duhamel gives the following formula for Opodeldoc, which, with a slight difference, is the one proposed by the Committee of Revision of the U. S. Pharmacopœia. It possesses the advantage over the present preparation, of not coagulating, as does, invariably, the officinal preparation.

CAMPHORATED TINCTURE OF SOAP.

(*Soap Liniment,*)

| | |
|---|---|
| Take of Soap, in shavings, . . . | 16 oz. |
| Camphor, . . . . . | 8 oz. |
| Oil of Rosemary, . . . | 2 fl. oz. |
| Alcohol, . . . . . | 7½ pints. |
| Water, . . . . . . | ½ pint. |

Dissolve the soap in water, by heat; then mix with it the alcohol in which the camphor and oil have previously been dissolved: lastly, filter.

---

### THE HYSTERIC DIATHESIS.

BY DR. S. ASHWELL.

"It is of great practical utility to remember that, where the hysteric diathesis really prevails, recoveries sometimes occur from states in which all hope has been laid aside. Thus, paralysis and difficulty of swallowing, and great debility, are extraordinarily recovered from; and occasionally when phthisis and the emaciation supposed to be its direct result have reached an apparently hopeless point, the patient most singularly and inexplicably begins to recover. I have sometimes thought that an impression on the mind of the sufferer of the certainty of a fatal result, if the disease persisted, was the first link in the chain of events, marking a gradual restoration. I am, too, quite certain that the progress of phthisis is often slower in the hysterical than in any other class. These and other considerations establish the extreme importance of an accurate and comprehensive knowledge of the symptoms and varying aspects of hysteria."

The treatment of the disease is divided into that applied to hysteria dependent on plethora, or debility, and on gastro-intestinal disorder, but nothing possessing the charm of novelty is presented to us, and Dr. Ashwell, like all other writers and physicians of the day, is obliged to treat symptoms and employ the same means as in times past.—*Provincial Medical Journal*, Sept. 16, 1843.

### URINE IN PNEUMONIA AND PLEURO-PNEUMONIA.

BY DR. F. SIMON, OF BERLIN.

In a case of pneumonia, and in another of pleuro-pneumonia, a species of urine was observed, which was remarkable at once for its extremely peculiar properties, the same in both cases, but deviating from the normal properties of urine, and also in this respect, that, as Dr. Schönlein ascertained, its appearance coincided accurately with the occurrence of resolution of the inflammation. In the first case of pneumonia, the urine during the violent inflammatory stage was dark, very acid, and unaccompanied by any sediment; at the time of resolution it was pale and neutral. One morning I found the urine of this patient yellow, neutral, and with a sediment of white crystals perceptible to the naked eye; the microscope instantly showed, by the remarkably beautiful form of the crystals, that they consisted of the triple combination of magnesia, ammonia, and phosphoric acid. The peculiar character of the urinary fluid itself surprised me; it was completely neutral, and every acid, even dilute acetic acid, produced in it a white precipitate, so that the first instant I suspected the presence of a caseous matter, but soon convinced myself, however, that this was not the case, for when I allowed a part mixed with hydrochloric acid to stand for some time, a precipitate was formed of beautiful, almost colorless uric acid crystals; these were also formed when I heated a part with acetic acid, and let it stand for some time. Alcohol formed a rather considerable precipitate, which was washed on a filter with alcohol; a portion of this precipitate was abstracted by warm water, which, on evaporating the water, remained behind; this was almost perfectly consumed on a platinum dish; when rubbed up with caustic potash it developed ammonia; heated with nitric acid, the presence of a great quantity of uric acid was ascertained. Whatever portion of the precipitate caused by alcohol was not dissolved in warm water, was readily taken up by hydrochloric acid, and again precipitated from the acid solution by ammonia; with the microscope I recognised this precipitate occasioned by ammonia as ammonio-magnesian phosphate. From this it results that the white precipitate occasioned in the urine by any acid, was uric acid, which was found dissolved in the urine in so extraordinary a manner combined with ammonia—a circumstance which, in my opinion, has not yet been noticed up to the present moment.

In the second case of pleuro-pneumonia, which occurred at a subsequent period in Dr. Schönlein's clinical wards, a species of urine was voided at the time of the resolution, which corresponded in every respect with that just described, especially with respect to the remarkable property now mentioned; here, too, we had the beautiful crystalline sediment of triple phosphate of magnesia, and the precipitability of the urine by any acid. Two important questions arise here, one of which may be easily answered by accurate observation—namely, whether this peculiar phenomenon in the urine is connected with the process of resolution of the inflammation in the respiratory organs? The second question is probably not so readily answered—namely, what sort of connexion this is? We may content ourselves at once with the solution of the first question, which appears of sufficient importance for the prognosis, and for which every practical physician will find an occasion at the sick bed. We may observe, that the phenomenon here remarked was observed in the urine for three or four days, and that in both cases a recovery took place.—*Prov. Med. Jour.*, Sept 9, 1843.

### DR. CHRISTISON ON THE NEW MODE OF DETECTING ARSENIC.

A short time since Professor Reinsch proposed an entirely new method of detecting arsenic, which consists in acidulating any suspected fluid with hydrochloric acid, and heating a thin plate of bright copper in it, upon which the arsenic is deposited in a thin metallic crust, and then separating the arsenic from the copper, in the state of oxide, by subjecting the copper to a low red heat in a glass tube. Organic fluids and solids may be prepared for this process by boiling them for half an hour with a little hydrochloric acid, solid matters being cut into small shreds, sufficient water being added to let the ebullition go on quietly. Continue the boiling until the solids are dissolved, or reduced to minute division. Nothing can be more easy than the method of Reinsch. It is also exceedingly delicate; for it will detect a 250,000th part of arsenic in a fluid, and it does not leave any arsenic in the subject of analysis which can be detected by any other means, even by the delicate process of Mr. Marsh. I have lately employed it as the means of furnishing irrefragable evidence in criminal inquiries.

The separation of arsenic upon copper, from a solution, by means of hydrochloric acid and heat, is a new fact in chemistry; and the experiment furnishes a test so far, that, if the copper be not tarnished, arsenic cannot be present. But Reinsch's discovery cannot be regarded as a positive test, because, as he himself has pointed out, bismuth, tin, zinc, and, above all, antimony, will, under the same circumstances, yield a coating to copper sufficiently similar to render it necessary that the deposit be examined otherwise than by the eye only. Reinsch's process, however, is of far greater value than if it had merely presented a new test for arsenic. It constitutes the easiest and most secure mode of so separating arsenic from complex mixture as to enable experimentalists to apply to the metal any of the tests for arsenic already known; and, in my opinion, no method of testing for it approaches the following in conclusiveness. Cut the copper on which the arsenic is deposited, into small chips, so that they may be easily packed at the bottom of a small glass tube. Apply a low red heat.—A white crystalline powder sublimes, in which, in the sunshine, or with a candle near it, a magnifier of five powers will show the equilateral triangles composing the facets of the octaëdral crystals which are formed by arsenious acid when it sublimes. Sometimes the three equal angles, composing a corner of the octahedron, may be seen by turning the glass in various directions. If triangular facets cannot be distinguished, owing to the minuteness of the crystals, then shake out the copper chips, close the tube with the finger, and heat the sublimed powder over a very minute spirit-lamp flame, chasing it up and down the tube until crystals of adequate size are formed. Next, boil a little distilled water in the tube over the part where the crystalline powder is collected, and, when the solution is cold, divide it into three parts, to be tested with ammoniacal nitrate of silver, ammoniacal sulphate of copper, and sulphuretted hydrogen, either in the state of gas or dissolved in water. I am surprised that, during the last four or five years, neither Orfila, nor M. Lassaigne, nor Liebig, nor Mr. L. Thomson, nor Mr. Watson, nor Mr. Marsh himself, nor any other experimentalist, excepting in Scotland, has thought of applying, as a test of an arsenical crust, the conclusive process described above, and first suggested to me in 1826 by the late Dr. Turner, which consists in converting the metal into the oxide in such a way as to allow the form of its crystals to be determined. The method has been in constant use in medico-legal researches in Scotland. Yet, what other method is so satisfactory? What substance, other than arsenic, yields the white sublimate with triangular facets, or leaves the substance in such subjection to so many excellent tests?

In boiling substances in the weak hydrochloric acid, a decided excess of acid must always be present,—two fluid drachms to every eight ounces of liquid; but if the matter be animal texture in decay, much more acid may be necessary, owing to the presence of ammonia, which gradually neutralizes the acid as the solution goes on. Filtration of the fluid after the acid has acted sufficiently seems advisable, otherwise organic particles may attach themselves to the copper, and give rise to empyreuma when the metallic arsenic is driven off by heat. Where the arsenic in the fluid is supposed to be small, nearly half an hour should elapse before the copper, or copper-leaf, is removed. Before applying the sulphuretted hydrogen as a test to the solution of the sublimed oxide, the solution must be acidulated with hydrochloric or acetic acid. In every case the whole process should be applied in the first instance to distilled water, acidulated with the hydrochloric acid to be employed afterwards; and if the copper be tarnished, a purer acid must be obtained, or the copper must be subjected to the subsequent steps of the process, in order to ascertain whether the tarnishing be occasioned by arsenic or not.

I have successfully employed the preceding method in two medico-legal cases, where the bodies had been buried for four months, and I consider that it must soon supersede the beautiful but much more elaborate method of Marsh.—*Lancet, from Lon. and Edin. Jour. of Med. Science.*

---

### CASE OF DISLOCATION OF THE ODONTOID PROCESS OF THE AXIS, AND ITS EFFECTS UPON THE NERVOUS FUNCTIONS.

An unmarried woman, twenty-one years of age, and who had ever previously enjoyed good health, became, at the commencement of 1841, suddenly subject to stiff-neck, which she attributed to having taken cold. She could certainly turn her head, but it was habitually deflected to the left side. While this continued to be the case, at the beginning of February a young man, in a frolic, having taken her head in both hands, turned it hastily towards the opposite side. Immediately the patient felt a sharp pain in the nape of the neck; she was afterwards unable to turn her head to the right side, and deglutition was impeded from that moment. A hard and insensible tumour now began to grow in the posterior cervical region. About the middle of March difficult emission of urine and sleeplessness supervened, and the movements of the left arm and of the fingers of the right hand were enfeebled, as were those of the lower extremities. The difficulty of deglutition often provoked cough; obstinate constipation came on. No acute pain was felt while the head was kept in one position, except occasionally at the back of the neck, where a hard tumour, insensible on pressure, existed in the middle line, opposite to the articulation of the two first cervical vertebræ. The slightest motion of the head were, however, very painful, and much dreaded by the patient, who sank in the course of increasing paralysis.

At the necropsy the odontoid ligament was found broken and disintegrated, though without any signs of suppuration. The odontoid process was not organically injured any more than any other part of the axis or the atlas; it was found to have compressed and caused excessive softening of the anterior fasiculi of the spinal cord, while the posterior columns displayed little alteration. It should be particularly noticed, in corroboration of the views of Sir C. Bell, &c., that the phenomena exhibited during life in this case, were in perfect accordance with those which would be *a priori* deducible from the post-mortem appearances, agreeably with the doctrines of that physiologist,—*the power of motion had been much more affected than sensation.—Ibid, from Gazette Medicale.*

### TREATMENT OF CEPHALALGIA.

Dr. R. Howard prescribes, with success, for nervous and various other headachs, a mixture of a drachm of acetic acid, an ounce of compound tincture of cardamoms, and four ounces of some convenient vehicle, of which he directs a mouthful to be taken every twenty minutes.—*Lancet.*

### UN-UNITED FRACTURE OF THE LEG SUCCESSFULLY TREATED WITH IODINE.

John Lawes, a sailor, of sallow complexion and light hair, aged thirty years, was admitted into the London Hospital, under Mr. Scott, Oct. 8th, with an un-united fracture of the lower fourth of the left tibia and fibula, ligamentous structure merely forming the bond of connection between the upper and lower portions of the two bones. The angle of junction was 130° from before backwards; the heel elevated about an inch beyond the opposite heel; the leg slightly flexed; and the apex of the outer malleolus prominent. The great toe pointed downwards and forwards. On making extension from the foot, the lower part of the leg could be brought more nearly into a line with the upper, owing to the yielding of the elastic medium.

He stated that about two years ago while clinging to the wreck of the Lord William Bentinck, in the East Indies, a large log of wood was brought by the waves into violent collision with the left leg, causing a fracture; that he was immersed up to his middle for ten hours in the water; and that considerable numbness succeeded in the lower extremities and lasted for three or four days after he had been conveyed on shore.

He was placed under the customary treatment for fifteen months, part of which time he spent in the Bombay Infirmary, and the remainder in the Queen's Depôt, from which latter establishment he was discharged as incurable.

He arrived in England about three weeks prior to admission. His constitution had been much enfeebled during the voyage, which circumstance he mainly attributed to his unavoidable inactivity during it.

He was immediately placed on a fracture-bed so constructed that permanent extension could be maintained from the foot (which was made a fixed point) by the weight of the pelvis acting through the medium of the femur on the upper part of the tibia. Generous diet and a tonic course of medicine were exhibited.

On Oct. 28, the extension having produced no material alteration in the affected leg, the tendo Achillis was divided, and a weight suspended by a bandage passed round the leg, immediately above the seat of fracture. Tincture of iodine was locally applied daily for the course of a month, at the end of which time an extensive callus had formed, of considerable firmness. The frequency of its application was now diminished to two or three times during the week, but persisted in for three months longer, when he was taken down from the apparatus and placed in the horizontal posture. The foot had assumed its natural position, the only deformity being a slight bow inwards at the place of union. He remained in the hospital three months longer, and a few days before he left walked a distance of nine miles without inconvenience or difficulty, and with the assistance of a single crutch, the use of which he soon dispensed with.

*London Lancet*, Sept. 16, 1843.

### *Some Observations on the Statistics of the Vibrio Humana. (Trichina Spiralis.)* By Dr. Knox.

In the Spring of 1836 a case occurred in the Practical Rooms, Old Surgeons' Hall, of the occurrence of this very curious entozoon infesting the human muscles. Numerous specimens of the worm and its sac were examined with great care by my brother and by myself, and the attention of the profession here was very generally directed to the case. The memoir was published in the July number of the Edinburgh Medical and Surgical Journal of the same year, viz. 1836. Since then, *one case only* has occurred in my Practical Rooms, viz. in October, 1839, in almost every respect so much resembling the first, that it would be a waste of time to give any details respecting it. Now in the interval more than a hundred persons had been examined anatomically, and with such care as to render it nearly impossible that the presence of the worm could have escaped observation had it really occurred. Again, the comparative rarity of its occurrence in Scotland with what has been stated to be the case in England or in Ireland, induced me to address a note to my anatomical colleagues here, and from them I have very politely received the following answer to my queries.

Dear Sir,—I have not met with an instance of the very curious entozoon (the vibrio humana, or trichina spiralis of Farre) discovered by Messrs. Hilton and Paget, of London, although I have turned my attention to it. The number of bodies which have been dissected in my rooms, and under my superintendence, amounts to one hundred and forty-three.—I am dear sir,

Yours faithfully,
P. D. Handysides.

Feb. 6, 1840.

My Dear Sir,—In answer to your inquiry, I beg to state that I have seen but one subject in the muscles of which the trichina spiralis was developed.—The subject was a female about fifty years of age, and of rather spare habit: the muscles were pale and soft. The trichinæ were very numerous, at once detected with the naked eye, and contained, as usual, in a minute white cyst. I regret now that I did not take notes of all the muscles in which they were contained. I have superintended the dissection of between two and three hundred subjects, and this is the only case in which they were found.

I remain, my dear sir,
Yours sincerely,
Alexander S. Lizars.

Feb. 13, 1840.

In addition to these notes from Drs. Handysides and Lizars, Mr. Mackenzie, who superintends Dr. Monro's Practical Rooms, has had the kindness to make me a lengthened verbal communication on the subject. He assures me that since the publication of my memoir the matter had received from him every attention, and he feels assured, that if any case had occurred in his Practical Rooms it could hardly have escaped observation; yet no case up to this date (February, 1840,) had ever occurred to him. Mackenzie comes therefore to the same conclusion as I and my other anatomical friends in town here have arrived at, viz. that the occurrence of the vibrio humana is very rare in Scotland, since of about five hundred persons examined anatomically there had occurred but *three cases.* This of course refers more especially to the poorest classes of society. What the average may be in the wealthier classes may, perhaps, be never ascertained.—*Lon. Med. Gaz, Sept.* 1.

*Some Account of the Epidemic of Scarlatina, which prevailed in Dublin from* 1834 *to* 1842, *inclusive; with Observations.* By HENRY KENNEDY, A. B.

The little work before us contains an account of a severe epidemic visitation of scarlatina, written by a practical man, who describes the symptoms he has himself witnessed, and the effects of remedies which he has enjoyed ample opportunities for putting to the test. The importance of the subject will be readily admitted, when we find, as our author tells us, "that in England, in the year 1840, the deaths from scarlatina alone amounted to 20,000; and supposing the rate of mortality to be the average, which, in a very extended scale, is now found to be about 6 per cent., this would show that in the one year there had been upwards of 330,000 cases—a number which, unless we had figures for it, could scarcely be believed.—The same method of calculating, too, shows that at the present moment there are upwards of 500 cases occurring weekly in London, as may be seen from the pages of the 'Medical Gazette.'"

The symptoms, pathology, and treatment, are detailed in succession, and illustrated by a good selection of cases. One of tne most remarkable symptoms met with in the severer cases was the occurrence of a diffuse cellular inflammation of the external parts of the neck and throat, followed by unhealthy suppuration and sloughing; and in no less than three cases death occurred from hæmorrhage caused by sloughing of the parieties of the internal jugular vein.—Sloughing of the mucous membrane of the throat, however, was very rare as a cause of death.

We will present to our readers a few points which we noted, for our own information, from Mr. Kennedy's observations on the effects of remedies.

*Emetics.*—A teaspoonful or two of mustard the most serviceable, at the beginning of the attack, to rouse the patient from a state of depression; *nothing allayed obstinate vomiting better;* he gave it also at any period of the disease when there was high fever and incessant restlessness. *Warm bath* frequently useful in allaying irritation when the vital powers admitted of it. *Cold and tepid ablution* almost always beneficial. *Cold affusion* was not much resorted to, but, when confined to the head, was found useful for violent raving and restlessness. *Blood-letting* very seldom admissible; leeches to the throat, or mucous membrane of the nose, occasionally brought great relief to head or throat symptoms; but, on the whole, the type of the epidemic entirely forbad depletion.—*Diluents*, copiously administered, the author believes to be of very essential importance. *Stimulants* required almost constantly; they could, throughout the whole epidemic, be administered with much greater certainty of benefit than in any analogous disease: wine and carbonate of ammonia preferred. *Blisters* dangerous from tendency to slough. *Purgatives* but little required, and only to be given with extreme caution. *Opium* the author "found a most valuable medicine, and one which holds out better prospects of saving life in some of the more serious kinds of scarlatina than any other remedy." He gave it, first, for the relief of great depression, weakness, and sleeplessness, attending typhoid symptoms in adults; secondly, for cases in which head symptoms, raving, and so forth, were prominent, and neither relieved by the appearance of the eruption, nor capable of bearing bleeding; thirdly, in cases of diarrhœa; but here it was almost powerless. For the *sore throat*, a few leeches, warm poultices, but, above all, a strong solution of nitrate of silver brushed over the internal surface. For the *diffuse external inflammation*, poultices and keeping up the strength. He saw no cases of recovery from this in children in which suppuration did not occur.—*Provincial Medical Journal,* Oct. 7, 1843.

TREATMENT OF HEMICRANIA AND TIC DOULOUREUX BY CAUTERIZING THE PALATE.

BY M. DUCROS, OF MARSEILLES.

In the most intense hemicrania, and in the most obstinate *tic douloureux*, whether fronto-facial or temporo-facial, the pain disappears almost instantaneously on the application of ammonia at 26°, to the palatine arch, by means of a [camel's-hair] brush; the brush being allowed to remain on the part till a copious flow of tears has been excited. I have tried this for the last three months in a very great number of cases, and the pain has always ceased. If the pain returns, a fresh application again produces a cessation of the neuralgia.—*Lon. Med. Gaz., from Gazette Médicale.*

CREAM OF TARAXACUM.

Cut the fresh roots of dandelion, freed from any adherent earthy matter, (previously washed and slightly scraped,) into transverse slices. Sprinkle any quantity of these, while moist, slightly with spirit of juniper, and express them in a tincture-press. The cream thus expressed will keep any reasonable time for the purposes of the practitioner in the hottest weather.—The dose, a table-spoonful, or more, twice or thrice a-day, will probably produce two or more diurnal biliary evacuations.

It may be diluted, and put up in the form of draughts, with any of the diuretic waters or infusions, or with a solution of cream of tartar.—*Lon. Lancet.*

LITHOTRITY AND LITHOTOMY IN CHILDREN.

The operation, I may say, in conclusion, is not well adapted to young subjects. The parts in them are excessively irritable, and the stones are, in the majority of cases, excessively hard. The fragments are got rid of with difficulty, and altogether I firmly believe that the operation of lithotomy is much to be preferred for young patients. I venture to express this opinion after having had ample experience. The operation of cutting children for stone, I may add, is unattended, in the hands of an experienced and expert surgeon, with risk of any kind, and, indeed, the mortality is not, or should not be, worth noticing; say one death in some hundreds of cases.—*Clinical Lecture by Mr. Liston. London Lancet.* Oct. 28, 1843.

RAYER has sought in vain for a case where the *kidney* can remove absorbed pus from the body.

